# A link between high serum levels of human chorionic gonadotrophin and chorionic expression of its mature functional receptor (LHCGR) in Down's syndrome pregnancies

**DOI:** 10.1186/1477-7827-3-25

**Published:** 2005-06-21

**Authors:** Subhasis Banerjee, Alan Smallwood, Anne E Chambers, Aris Papageorghiou, Hugues Loosfelt, Kevin Spencer, Stuart Campbell, Kypros Nicolaides

**Affiliations:** 1 Harris Birthright Research Centre for Fetal Medicine, King's College Hospital Medical School, Denmark Hill, London SE5 9RS, UK; 2 INSERM U135, Biochimie hormonale, CHU de Kremlin-Bicêtre, Bat Paul Broca, 3e niveau 78 avenue du général Leclerc, 94275 Le Kremlin-Bicêtre, France; 3 Endocrine Unit, Clinical Biochemistry Department, Harold Wood Hospital, Gubbins Lane, Romford RM3 0BE, UK

## Abstract

Human chorionic gonadotrophin (hCG) is released from placental trophoblasts and is involved in establishing pregnancy by maintaining progesterone secretion from the corpus luteum. Serum hCG is detected in the maternal circulation within the first 2–3 wks of gestation and peaks at the end of the first trimester before declining. In Down's syndrome (DS) pregnancies, serum hCG remains significantly high compared to gestation age-matched uncompromised pregnancies. It has been proposed that increased serum hCG levels could be due to transcriptional hyper-activation of the *CGB* (hCG beta) gene, or an increased half life of glycosylated hCG hormone, or both. Another possibility is that serum hCG levels remain high due to reduced availability of the hormone's cognate receptor, LHCGR, leading to lack of hormone utilization. We have tested this hypothesis by quantifying the expression of the hCG beta *(CGB)* RNA, *LHCGR* RNA and LHCGR proteins in chorionic villous samples. We demonstrate that chorionic expression of hCG beta *(CGB)* mRNA directly correlates with high serum hCG levels. The steady-state synthesis of *LHCGR* mRNA (exons 1–5) in DS pregnancies was significantly higher than that of controls, but the expression of full-length *LHCGR* mRNA (exons 1–11) in DS was comparable to that of uncompromised pregnancies. However, the synthesis of high molecular weight mature LHCGR proteins was significantly reduced in DS compared to uncompromised pregnancies, suggesting a lack of utilization of circulating hCG in DS pregnancies.

## Introduction

The incidence of aneuploidy in human pregnancies is unusually high (1–2%) compared to other mammals [1]. Monosomies and trisomies together account for 35% of clinically detected spontaneous abortions (6–20 wks of gestation), stillbirth (4%) and most importantly, are the leading cause of developmental disability and mental retardation of those surviving such pregnancies [2-4]. Of all the genetically compromised pregnancies, Down's syndrome (Trisomy 21, T21) is the most frequent (1/700 live births [5]). The Edward's (Trisomy 18, T18) and Pautau's (Trisomy 13, T13) syndromes are considered relatively rare pregnancy disorders with a prevalence at birth of 1 in 7000 and 29000, respectively [6,7].

Genetically, 89–95% of Down's syndrome (DS) patients carry an extra chromosome 21 (chr 21) which arises due to meiotic nondysjunction and is usually inherited from the mother [1]. About 1–2% of DS patients have genetic mosaicism (nondysjunction following fertilisation in early embryos), while 3–4% of cases are due to translocation of chr 21 to another autosome, usually chr 14 [8]. In addition to the characteristic variability in mental retardation, physical and facial features, congenital heart and gastro-intestinal defects, the DS patients are also susceptible to leukaemia and Alzheimer's-like dementia [9-11].

The chromosomal abnormalities in DS and other trisomic pregnancies are very often associated with increased or reduced levels of proteins, growth factors and hormones in the maternal blood compared to those of normal pregnancies. For example, in DS pregnancies (11–14 wks of gestation), the serum human chorionic gonadotrophin beta (hCG-β) and pregnancy-associated plasma protein-A (PAPP-A) concentrations tend to be high and low, respectively [12].

Human chorionic gonadotrophin (hCG) is the key reproductive hormone regulating human pregnancy. It is a member of the family of glycoprotein hormones that includes luteinizing hormone (LH), follicle stimulating and thyroid stimulating hormones, each member of which functions through the formation of a non-covalent heterodimer from two subunits, α and β.

In human placenta hCG is primarily produced by syncytotrophoblasts and to a certain extent by extravillous cytotrophoblasts [13]. One of the earliest endocrine roles of hCG is to sustain the corpus luteum which must produce enough progesterone to establish pregnancy at the outset. In addition, hCG facilitates trophoblast differentiation, remodeling of the uterine epithelium and stroma (decidualization) and endometrium for implantation, invasion of the maternal spiral arterioles, and angiogenesis by acting on vascular smooth muscle and endothelial cells [14]. In normal pregnancies, detectable levels of hCG begin to appear in the maternal circulation at about 2–3 wks after conception, and reach their peak at ~11–13 wks before declining significantly in the later stages of pregnancy. Indeed, high serum hCG levels at mid-late pregnancy have been associated with pre-eclampsia, intra-uterine growth restriction and Down's syndrome (DS) [15-18].

The hCG hormone transduces signals by binding to its specific LH/hCG receptor (LHCGR) expressed on surface of the cell. Since hCG and LH receptors are identical, it is often referred to as the LH/hCG receptor (LHCGR) and is encoded by a single copy ~70 Kb *LHCGR *gene, located on human chromosome 2p21 [19]. This receptor is structurally very similar to two other hormone receptors (thyroid stimulating and follicle stimulating hormone receptors). The *LHCGR *gene has 11 exons and codes for multiple alternatively spliced species (at least 6) of mRNA. These different mRNA transcripts are initiated at multiple sites spanning a region more than a kilobase upstream of the first exon [20].

On the basis of structure and topology, LHCGR is a member of the rhodopsin/β-adrenergic receptor superfamily of G protein-coupled receptors. Agonist (hormone) binding to LHCGR allows dissociation of membrane-bound cognate G proteins that regulate phospholipase C, adenylyl cyclase and ion channels which in turn control cellular inositol phosphates, cAMP, Ca^+2 ^and other secondary messengers [21,22].

LHCGR is a 701 amino acid residue protein containing three distinct domains: an unusually large (340 residues) N-terminal extracellular domain which binds hCG, a serpentine transmembrane (TM) region containing seven TM repeats connected by three extra- and intracellular loops, and a C-terminal tail. The predicted relative molecular mass (M_r_) is ~75 K, or higher, depending upon the level of glycosylation [23].

Moreover, alternatively spliced mRNAs produce several truncated intra-cellular protein isoforms which have ligand binding capacity but are ineffective in transducing signals [24]. The functional significance of all isoforms remains to be established. However, the accessibility of the M_r _85–95 K species to surface biotinylation, protease and glycosidase (neuraminidase), suggests that they have ligand binding and signal transduction capacities. On the other hand, the M_r _65–75K proteins contain high-mannose type side chains which are susceptible to endoglycosidase H, and are immature and intracellular [25-27]. The high relative molecular mass 165–200K group is thought to be a dimer of the mature functional receptor [28]. Interestingly, smaller species of LHCGR proteins (M_r _45–51K) can be detected in tissues or cells transfected with cDNAs [25-27,29].

Natural missense [30] and deletion mutations [31] of the human LH receptor have been reported to be associated with elevated serum LH levels in these patients. Similarly, the circulating LH concentration remains high [32,33] in mice carrying a homozygous deletion of *Lhr *gene (*Lhr*^-/-^). Moreover, the lack of functional cytokine receptor expression, due to natural mutations of the IFN-γ receptors 1 and 2, has been directly linked to high serum IFN-γ levels in patients suffering from infectious diseases [34,35]. These reports prompted us to investigate whether increased serum hCG levels in DS pregnancies could be linked to the expression of its functional receptor in chorionic villi.

## Materials and methods

### Placental tissues and chorionic villous samples

This study was approved by the local ethics committee of King's College Hospitals, London, UK and written consent was obtained from patients before the collection of samples. Placental tissue was obtained from patients undergoing termination of pregnancy with gestational age range of 7–12 wks, after vaginal delivery or caesarian section. Chorionic villous samples (CVS) were collected in a Petri dish and material not required for genetic analysis was washed in Ca^+2^/Mg^+2^-free PBS (Invitrogen, Carlsbad, CA, USA) and stored in 500 μl RNA Later (Ambion, Huntingdon, UK), held at 4 C overnight before long term storage at -20°C.

### RNA extraction and cDNA synthesis

Depending upon the availability, up to 30 mg of CVS was extensively washed in PBS, homogenized in 500 μl Trizol (Invitrogen) with a tissue grinding pestle (Anachem, UK). Subsequent RNA extraction, DNase I treatment (Sigma, St Louis, MO, USA), cDNA synthesis were exactly as described previously [36,37]. In some cases, a Qiagen RNA extraction kit (Qiagen, West Sussex, UK) was used and RNA was stored at -70°C in water or ethanol.

### Quantitative PCR

Quantitative PCR was performed using the Light Cycler RNA amplification system in glass capillaries and a fluorescence-based hybridization detection format (Roche Diagnostics GmbH, Mannheim, Germany) as described [38]. Briefly, the assays were carried out in duplex where both the experimental sample and an internal control (*ACTB *and *HPRT*) were run in the same reaction. The reporters LC-Red 640 and LC-Red 705 were employed to generate hybridization probes for experimental and internal controls and the amplification for each cDNA was recorded by dual color detection. A color compensation file was created according to the manufacturer instructions (Roche Diagnostics) and was compensated for PCR cycles in duplex during each run. In some experiments, the control reactions were run at the same time as the test samples under the same reaction conditions, but as single reactions. In order to establish that there was no cross contamination, negative controls (a full reaction without cDNA) were run in each experiment.

Cross-contamination was avoided by sequentially adding water, reaction master-mix (containing enzyme, Mg^+2 ^and PCR buffer; Light Cycler RNA amplification Kit, Roche Diagnostics), and cDNA to a final volume of 10 μl. The analysis mode set to quantification included initial denaturation at 95°C for 10 min followed by 40 cycles consisting of the following parameters for segments 1–3: Target temp, 95°C, 55°C and 72°C respectively; incubation time 10, 7 and 12 sec respectively; Transition rate 20°C/sec, 20°C/sec and 10°C/sec respectively, and a single acquisition mode at segment 2. PCR primers, hybridization probes and amplicon lengths are shown in Table 1. Data were automatically collected and filtered to remove background by the Light Cycler software which set the crossing point for all the different reactions against the standard curve. Data were transferred to Microsoft Excel for further analysis.

**Table 1:** Quantitative PCR primers, HUGO approved gene names, GenBank accession numbers, hybridization probe sequences and the length of respective amplicons.

Gene	Acc. No	PCR Primer	Hybridization Probes	bp
*ACTB*	XM_037235	F 5'-agc ctc gcc ttt gcc ga-3' R 5'-ctg gtg cct ggg gcg-3'	5'-ttg cac atg ccg gag ccg ttg-FL-3' 5'-LC Red 705-cga cga cga cgc cgg cga tat c-Ph-3'	178
*HPRT*	M31642	F 5'-atc aga ctg aag agc tat tgt aat gac ca-3' R 5'-tgg ctt ata tcc aac act tcg tg-3'	5'-aga ctt tgc ttt cct tgg tca ggc agt-FL-3' 5'-LC Red 705-aat cca aag atg gtc aag gtc gca agc-Ph-3'	230
*CGB *(hCG β)	NM_000737	F 5'-gac gca cca agg atg gag at-3' R 5'-gcg gta gtt gca cac cac ct-3'	5'-gtg tgc atc acc gtc aac acc acc-FL-3' 5'-LC Red 640-tct gtg ccg gct act gcc cca c-Ph-3'	251
*LHCGR *Ex 1–5	NM_000233	F 5'-tcg act atc act tgc cta cc-3' R 5'-gga gaa gac ctt cgt aac at-3'	5'-ttt gtc tga aat act gat cca gaa cac ca-FL-3' 5'-LC Red 640-aat ctg aga tac att gag ccc gc-Ph-3'	291
*LHCGR *Ex 11		F 5'-act tcc tta ggg tcc tg-3' R 5'-gtg atg acg gtg agg g-3'	5'-ggc tct atc tgc tgc tca tag c-FL-3' 5'-LC Red 640-cag ttg att ccc aaa cca agg g-Ph-3	303

### Cell culture, protein extraction from placenta and CVS, gel electrophoresis and Western blots

The HEK-293 cell line expressing N-terminal 362 amino acid residues of human *LHCGR *was kindly provided by Professor Axel Themmen, Erasmus Universty, Rotterdam, The Netherlands. The expression vector contained LHR extracellular domain (ECD, 1–362) fused to a tag peptide (YPYDVPDYA) from the hemagglutinin 1 (HA1) epitope of influenza virus and tetracycline (tet)-inducible promoter. Cells were grown exponentially in tet-free fetal bovine serum prior to overnight induction with tetracycline as recommended.

Protein extraction from cultured cells, placental villous tissues with T-PER (Perbio, Helsinborg, Sweden) and from CVS, following Trizol lysis were exactly as described previously [36] except the 50 mM Tris-HCl, pH 8.0 buffer was replaced by 25 mM HEPES-OH, 8.0. The total protein concentration in each extract was measured in duplicate (Lowry assay; BioRad DC substrates, BioRad, Hemel Hempstead, UK). Based on this estimate, approximately, 10–20 μg of total protein was loaded in each lane; for each CVS sample it was 10 μg per lane. The separation of proteins by SDS-polyacrylamide gels and Western blot analysis were as described [36-38]. Both 1% casein and 1% non-fat milk were equally effective blocking agents in Western blots.

The primary and secondary antibodies used were as follows: murine control IgG (Sigma) at a concentration of 1 μg/ml, protein A-sepharose purified anti-human LHCGR mouse monoclonal antibody (LHR-29) at a concentration 1 μg/ml, anti -β Actin, clone AC-15 (Sigma) and goat anti-mouse IgG (H+L) HRP-conjugated (Chemicon International Inc., CA, USA) at dilutions of 1 in 2000 and 1 in 5000, respectively.

### Hormone assays

Patient information and consent forms were given to each patient who came for ultrasound scan at HBRC, King's College Hospital. The venous blood (5 + 5 ml) was collected from those who consented (at 12–14 wks of gestation) with and without anticoagulant. Sera and plasma obtained by centrifugation (1500 rpm, 10 min at 4 °C) were aliquoted and stored at -20 °C.

Free hCG β and intact hCG were measured using the Brahms Kryptor (Brahms AG, Berlin, Germany) random continuous access immunoassay analyzer by a time resolved amplified cryptate fluorescence emission method. The performance of these methods have been described previously [39,40].

### Densitometry and data analysis

Densitometry of autoradiograms was carried out using a 1D-Multi Lane Densitometry program in an AlphaImager (1220v5.5, Alpha Innotech Corp. San Leandro, CA, USA) as described [36-38]. Scan data (experimental and β-actin control) were transferred to Microsoft Excel where the pixel density of each experimental lane was normalized to its corresponding β-actin value. Each experiment was repeated at least twice and average values for each data point were plotted. The means, standard deviations, variance (anova) for each data-set were computed using Analysis ToolPak (ATP) software. Values are shown as mean +/- SEM. A value for the level of significance (*P*-value) was calculated using the Poisson statistic. *P*<0.05 was considered significant. In experiments, where mRNA expression and the serum hormone concentration data were not normally distributed, the median values and 95% confidence intervals were calculated and the Mann-Whitney non-parametric U-test was employed to establish statistical significance.

## Results

The goal of this study was to examine the placental expression of the *LHCGR *mRNA and functional receptor protein expression with respect to serum hCG concentrations in Down's syndrome pregnancies. To achieve this, 1,152 CVS from high-risk pregnancies were collected. Of these, 58 were Down syndrome (DS, trisomy 21 [T21]), 22 Edwards's syndrome (trisomy 18 [T18]) and 12 were Patau's syndrome (trisomy 13 [T13]) confirmed by biochemical, molecular and cytogenetic analyses. The number of samples that contained sufficient tissue for RNA analysis was 41 for T21, 14 for T18 and 7 for T13.

### CGB (hCG β) and LHCGR genes are hyperactivated in Down's syndrome pregnancies

The hCG α subunit is synthesized in excess and is common to all members of this hormone family, whereas the hCG β subunit, which recognizes the cognate receptor, is specific for the hormone. Therefore, in order to evaluate the chorionic regulation of the hormone, the expression of hCG β mRNA synthesis was measured.

**Figure 1:**
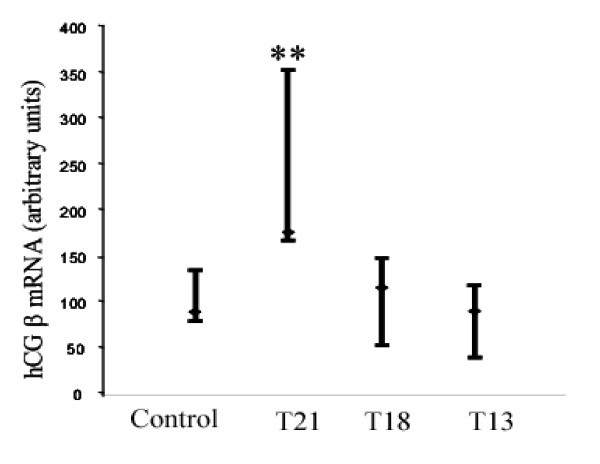
***CGB *(hCG β) expression in chorionic villous samples (CVS) from DS and other trisomic pregnancies. **Quantitative PCR analysis (Table 1) of the chorionic hCG β (*CGB*) mRNA expression in control (N = 24), T21 [DS, N = 41], T18 (N = 14) and T13 (N = 7) pregnancies. **P<0.01

The expression of hCG β (*CGB*) mRNA in CVS were assayed by quantitative real-time PCR (Q-PCR) amplification of cDNA. Since mRNA expression values in trisomic pregnancies exhibited a wide range of distribution, the 95% confidence interval limits and the median values in each pregnancy conditions were determined. Such analysis revealed that *CGB *(hCG β) gene expression in Down's syndrome CVS was significantly higher (*P *<0.001) compared to that of controls (Fig. 1). Moreover, hCG β (*CGB*) mRNA expression levels in T18 and T13 pregnancies were comparable to those of controls (Fig. 1).

**Figure 2:**
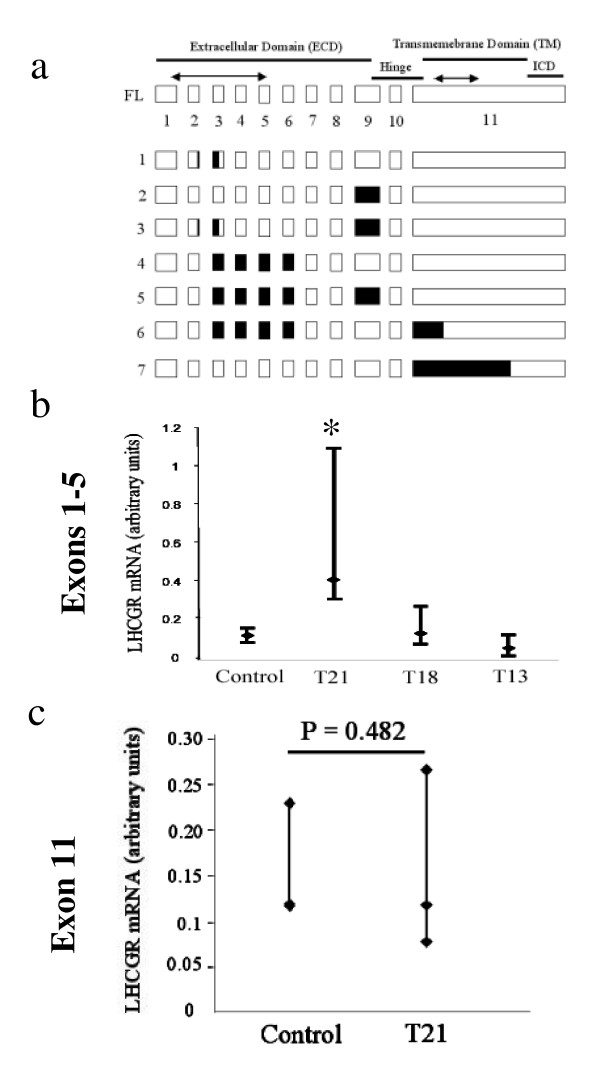
**The structure of LHCGR mRNA and expression in chorionic villous samples (CVS) from DS and other trisomic pregnancies. **a) the organization of exons 1–11 in full-length (FL) LHCGR mRNA (open boxes) and possible alternatively spliced isoforms 1–7 [41-43]. The sequences deleted from the isoforms are indicated by closed boxes. Regions of mRNA encoding the extracellular domain (ECD), transmembrane (TM) domain, the hinge region and the intra-cellular domain (ICD) are shown. The regions of cDNA (exons 1–5 and exon 11) amplified by Q-PCR are indicated by bidirectional arrows. b) chorionic *LHCGR *mRNA expression (exons 1–5) in control (N = 24), T21 [DS, N = 23], T18 (N = 8) and T13 (N = 3) pregnancies; c) chorionic *LHCGR *mRNA expression (exon 11) in control (N = 15) and T21 (N = 18) pregnancies. The median values and 95% confidence ranges of RNA expression in each pregnancy condition are shown; N = number of patient samples analyzed. *P < 0.05, **P < 0.01.

The structure of the full-length (FL) human *LHCGR *cDNA (exons 1–11), the coding region for extracellular domain (ECD), hinge region, transmembrane (TM), and intracellular domains (ICD) of the receptor, together with multiple alternatively spliced isoforms [41-43] are shown in Fig. 2a. The ECD alone has high ligand affinity [44] whereas, the TM and ICD are necessary for signal transduction [24]. The majority of isoforms exhibit deletion of exon 9 and 11 as observed in sheep [45], pig [21,27] and rat [46]. Deletion of coding sequences due to alternative splicing of isoforms 1–5 maintains an open reading frame, but in isoforms 6 and 7 frame-shift mutations are introduced resulting in stop codons at exon 11 and the potential production of soluble truncated receptor [42,43].

The *LHCGR *mRNA (exons 1–5) expression in DS pregnancies was significantly higher (*P*= 0.0501) compared to that of T18, T13 and uncompromised pregnancies (Fig. 2b). The *CGB *gene expression (Fig. 1) positively correlated with *LHCGR *exons 1–5 mRNA synthesis (correlation coefficient, r= 0.61). These results suggested that both the *CGB *and *LHCGR *genes were hyperactivated in DS placenta compared to expression levels in normal and other trisomic (T18 and T13) pregnancies. Notably, the quantitative increase in hCG β (*CGB*) /*LHCGR *mRNA production in the T21 group of pregnancies (Figs. 1 and 2b) was at least 3-fold higher than that observed for the T18 and T13 CVS.

The results described above showed a significant increase in *LHCGR *mRNA transcription in DS placenta compared to that of control pregnancies. However, as noted above, alternative splicing could give rise to mRNA variants that may not encode the full-length functional receptor. As a critical test of whether the quantitative increase in *LHCGR *(exons 1–5) mRNA in DS placenta truly reflects full-length receptor mRNA synthesis, the transcription of the 3' end of the gene (representing exon 11) was measured by Q-PCR using the same set of cDNA samples. The results (Fig. 2c) demonstrate that the chorionic expression of exon 11 in DS is comparable to that of control pregnancies, indicating that a significant population of *LHCGR *mRNA in DS placenta does not contain parts of exon 11.

In further attempts to measure the full-length *LHCGR *transcripts, we have tested three sets of custom-designed primer and probe to amplify exons 10–11 by Q-PCR. None of these were capable of amplification of *LHCGR*-specific cDNA whereas β-actin, *CGB*,*IFNGR1*,*IFNGR2*, *LIFR *and syncytin could be amplified by semiquantitative and Q-PCR. The *LHCGR *exons 7–9 could be amplified by Q-PCR from placental cDNAs obtained from early and late pregnancies. However, the signal intensity was reduced by at least 100-fold compared to that of exons 1–5 or exon 11. Therefore, the amount of cDNA in CVS samples was not sufficient for either semiquantitative or Q-PCR. To ensure that the light-cycler signals during exon 11 amplification were not due to DNA contamination, equivalent amount of the DNase-treated mRNA (not reverse transcribed) corresponding to experimental samples were tested. Amplification only occurred when cDNA was added to the Q-PCR.

### Serum hCG β and intact hCG (α and β) are significantly elevated at 11–14 wks gestation in Down's syndrome pregnancies

**Figure 3:**
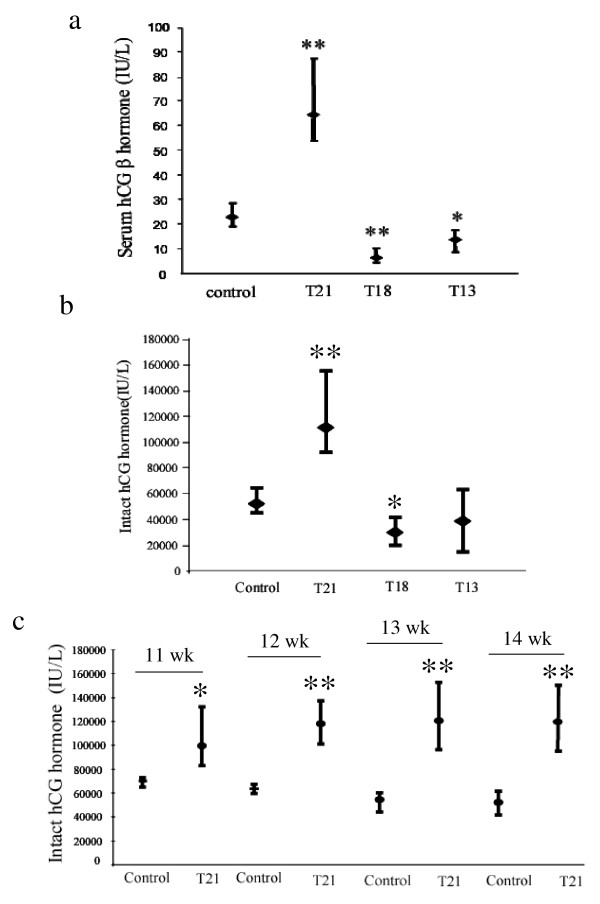
**Serum hCG β and hCG heterodimer concentrations in DS and other trisomic pregnancy conditions. **a) serum hCG β hormone concentrations in control (N = 18), T21 (N = 23), T18 (N = 7) and T13 (N = 4) pregnancies b) serum hCG heterodimer concentrations in control (N = 19), T21 (N = 15), T18 (N = 12) and T13 (N = 4) pregnancies; c) serum hCG heterodimer concentrations at 11 wk (control, N = 417; T21 N = 68), 12 wk (control, N = 417; T21, N = 148), 13 wk (control, N = 161 ; T21, N = 73), and 14 wk (control, N = 65 ; T21, N = 14) pregnancies; The median hormone concentrations and 95% confidence intervals for each data set are shown. N = number of patient samples analyzed. *P < 0.05, **P < 0.01.

Consistent with data from previous studies [12,17,39,40], serum hCG β levels in DS pregnancies were significantly higher (*P <0.01*) compared to that of uncompromised pregnancies. Moreover, serum hCG β levels were lowest in T18 (*P <0.01*) and were significantly low in T13 (*P <0.05*) compared to control serum samples (Fig. 3a). When adjusted for gestational age, the mean serum hCG β concentrations in DS were between 2 and 3.6-fold higher than those of normal pregnancies (12–14 wks). Serum hCG β levels positively correlated (r = 0.39) with hCG β (*CGB*) mRNA expression.

As noted above, the circulating hCG which transduces signals by binding to its receptor LHCGR is intact hCG heterodimer composed of both α and β subunits. In order to establish the relation between the circulating ligand hCG and its receptor expression, we next measured the hCG heterodimer concentrations in serum from normal and trisomic pregnancies. Such analysis revealed that serum hCG heterodimer concentrations in DS pregnancies (12–14 wks) was significantly increased (*P <0.01*) compared to that of control sera (Fig. 3b). The hCG heterodimer concentration in T13 pregnancies was comparable to uncompromised controls, but was significantly reduced (*P <0.01*) in T18 pregnancies (Fig. 3b).

These results suggest that circulating free β hCG and intact hCG hetrodimers are abundant at 11–14 wks of pregnancy in DS. The data shown in Fig. 3b were obtained from a limited number of serum samples from control (n, 18) and DS (n, 24) pregnancies predominantly at 12–14 wks of gestation. In order to further verify these data, the hCG heterodimer concentrations at 11, 12, 13 and 14 wks of pregnancy measured in sera from a large number of control and DS pregnancies as part of the previous studies [39,40] were compared. Such analysis revealed that hCG heterodimer concentrations in DS pregnancies were significantly higher than that of control sera at each time point tested (Fig. 3c).

### Western blot analysis to establish the specificity of the mouse monoclonal anti human LHR29 antibody

**Figure 4:**
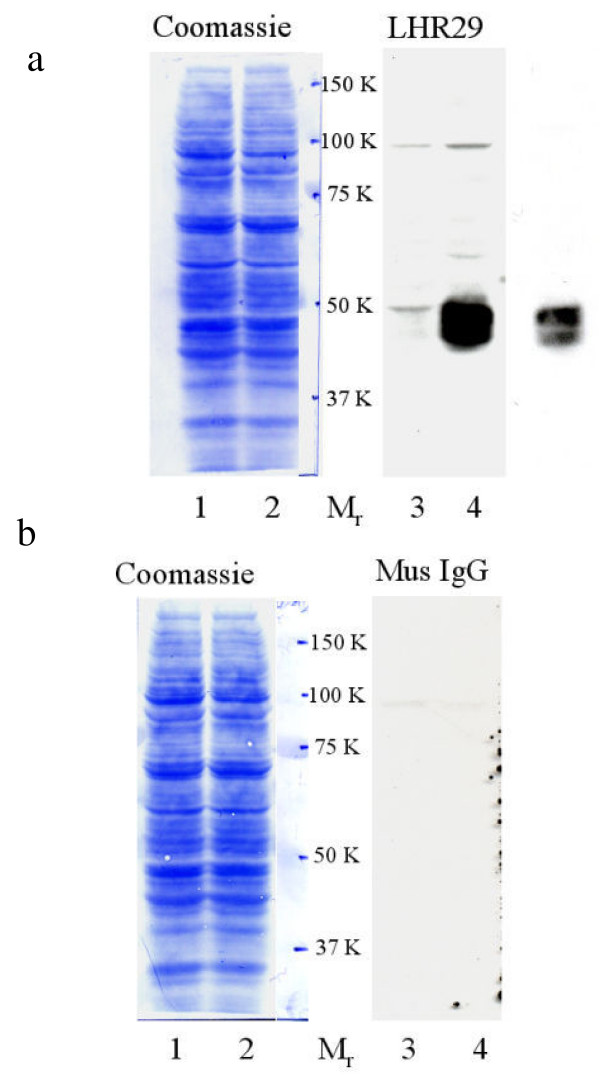
**The LHCGR extracellular domain (ECD) expressed in HEK-293 specifically reacted with human LHCGR mouse monoclonal antibody, LHR29. **HEK 293 cells (expressing human LHCGR ECD, amino acid residues 1–362) were grown exponentially in tetracycline-free fetal bovine sera in the absence (lanes 1 and 3 in a and b, respectively) and in the presence of tetracycline (lanes 2 and 4 in a and b, respectively). Each lane contains 25 μg of total protein separated via electrophoresis through 8% polyacrlamide SDS gels. Blots were immuno-reacted with antibodies (lanes 3 and 4 of a and b, respectively). A shorter exposure of lane 4 (a) is shown on the right hand side of the Fig. 4a. Following chemiluminescence detection, blots were stained with coomassie brilliant blue (lanes 1 and 2 of a and b, respectively).

LHR29 monoclonal antibody was originally obtained by immunizing mice with the purified recombinant human receptor extracellular domain (amino acids 75–406) expressed in *Escherichia coli*. The specificity of the antibody was verified by its ability to immunoprecipitate recombinant receptor and immunopurify ^125^I-hCG-receptor complexes from transfected cells, western-blot analysis with the immunogen, immunocytochemistry in cells transfected with either the cloned receptor or a mock vector. Patterns obtained by immunohistochemistry of human testis matched with results expected for a transmembrane receptor specific to Leydig cells ([47], and Axel Themmen, personal communication). In order to further verify the antigenic specificity of this antibody, the HEK 293 cell line expressing LHR ECD (1–362) was grown in the presence and absence of tetracycline. Extracted proteins were resolved via 8% SDS-PAGE, Western blotted, and blots were reacted with control mouse IgG or LHR 29 monoclonal antibody. In order to ensure that equal quantities of protein were transferred, the blots were stained with coomassie brilliant blue following chemiluminescence detection. The results (Fig. 4a, and 4b) demonstrate that the LHR29 monoclonal antibody specifically recognizes at least three (M_r _44–48K) tet-inducible species of LHCGR expressed *in vitro *(two bands appear to migrate as doublet). These three variants possibly reflect different levels of glycosylation. Moreover, these species (M_r _44–48K) are also recognized by anti-HA1 antibody in Western blots (Axel Themmen, personal communication), providing a further line of evidence that the LHR 29 antibody reaction is specific.

### Human placenta expresses at least six LHCGR protein variants

**Figure 5:**
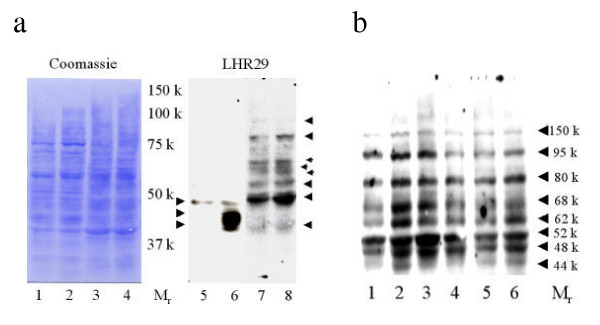
**At lease six LHCGR protein isoforms are expressed in human placenta. **a) The HEK 293 (LHCGR ECD 1–362) cells grown in the absence (lane 1), in the presence of tetracycline (lane 2), 7 wk (lane 3) and 10 wk (lane 4) of gestation placental tissues were lysed with detergent (T-Per) and extracts (10 μg of protein in each lane) were immuno-reacted with LHR 29 antibody; b) Placental tissues (10–14 wks gestation) were extracted with Tri-zol reagents, proteins (10 μ/lane) were separated by extended electrophoresis in 8% SDS-PAGE and the blot was reacted with LHR29 antibody.

In order to examine the LHCGR proteins produced in human placenta, the villous tissues obtained from 7 wk- and 10 wk-gestational age placenta were detergent extracted, reacted with non-specific control mouse IgG (not shown) and LHR29 in Western blots. To further control the experiment, the extracts from HEK293 (LHR ECD) were also incorporated and the blots were stained with coomassie blue following chemiluminescence detection. We detected (Fig. 5a) at least six major LHCGR variants ranging in molecular mass (M_r_) from 44K-95K (44K, 48K, 52K, 62–68K, 80K and 95K). These bands were also detected by LHR74 which recognizes LHCGR epitopes different from that of LHR29, but not when the primary antibody was murine IgG (data not shown). The Western blot patterns of human placental tissue obtained with LHR29 antibody were very similar to those described by VuHai-LuuThi *et al *in porcine testis [27,47]. Additionally, our results are fully consistent with the data more recently reported by Bukovsky *et al *in human tissues [28] using independent mouse monoclonal antibodies (anti-LHR mAb clone 3B5) and in LH-induced human M17 neuroblastoma cells [48] using a rabbit polyclonal antibody (raised against the N-terminal peptide sequence 15–38 of the rat LH/CG receptor).

The chorionic villous samples (20–30 mg tissues) are not sufficient for separate RNA and protein analysis. We have recently described a method where RNA, DNA and proteins can be quantitatively recovered from the same sample by Trizol extraction [36]. The results shown in Fig 5b demonstrate that the detergent extracted LHCGR protein variants from placenta (Fig. 5a) were identical to those observed with Trizol extracted proteins from human placenta. Indeed, the Trizol-extracted bands were somewhat sharper than the corresponding detergent extracted LHCGR variants in Western blot analyses.

### Expression of high molecular weight full-length LGCGR proteins is reduced in Down's syndrome pregnancies

**Figure 6:**
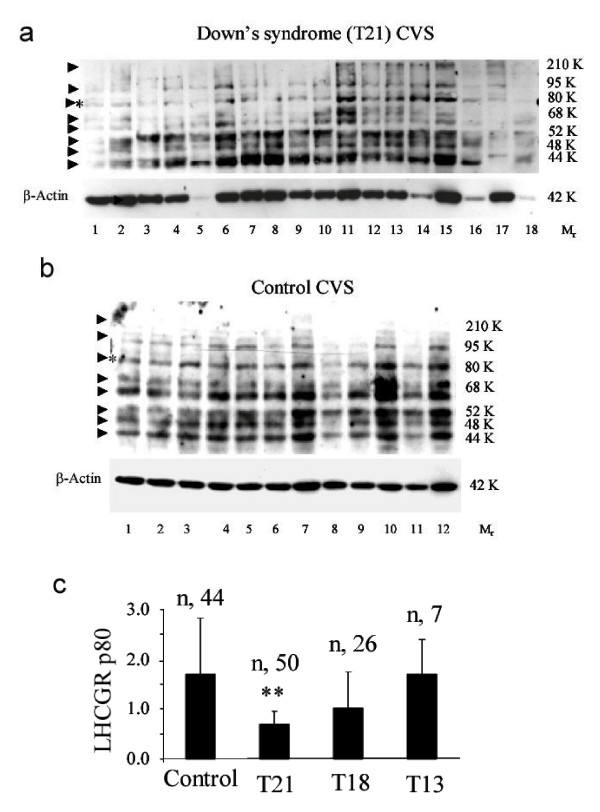
**The production of mature LHCGR isoforms in chorinic villi from Down's syndrome pregnancies are significantly reduced compared to that of controls. **The CVS samples were Tri-zol extracted to recover mRNA as well as proteins. Approximately 10 μg of total protein was loaded in each lane. The proteins extracted from DS (T21) CVS (a) and control CVS (b) pregnancies were resolved in 8% polyacrylamide-SDS gels, Western blotted and immunoreacted with anti-human LHCGR (LHR-29) monoclonal antibody. Blots were stripped prior to immunostaining with anti-β-Actin monoclonal antibody. The data shown in a) and b) were from the same experiment except that the control and DS proteins were separated in two gels at the same time. In order to compare the band intensity in different experiments, two known CVS samples in duplicate were incorporated in each experiment. The density of the 80K LHCGR and 42K β-actin bands served as references for quantitative analysis of the experimental samples. The relative migration of the isoforms is indicated by an arrow. The M_r _80K protein band (LHCGR p80), indicated by * in a) and b), well separated from the neighboring variants were scanned and c) the relative densities of the LHCGR p80 with respect to β-Actin in normal and trisomic pregnancies, n = total number of experiments carried out on protein samples in each condition. **P < 0.01.

In order to compare the LHCGR mRNA and protein expression both mRNA and protein samples were extracted from the same Trizol lysate. The LHCGR protein expression in control and genetically compromised CVS was examined by Western blot analysis and representative data from such analyses are shown in Fig. 6.

The M_r _80–110K LHCGR protein isoforms are thought to be the full-length functional receptor which is expressed on the cell surface and has ligand binding and signal transduction capacity [24,27]. In order to distinguish between the expression of full-length and other LHCGR isoforms, proteins in trisomic and normal CVS were separated by extended electrophoresis and LHCGR variants were detected by Western blot (Fig. 6a and 6b). A visual examination of the blots shows that that the mature isofoms (≥80K) were less abundant in DS CVS compared to control pregnancies. Moreover, the stoichiometric yield of the variants (M_r _44K-52K) in DS CVS (Fig. 6a) appears to be distinctly different from that of control CVS (Fig. 6b). For direct comparison, the full-length functional isoform was quantified by densitometry of the M_r _80K bands with respect to β-Actin expression in each lane. Each sample including the reference in duplicate (control CVS extracts) was analysed in at least two independent experiments and the mean band density was used as a measure of expression for each sample. Placental expression of LHCGR (M_r _80 K) was lowest in DS (*P*<0.01), remained unchanged in T13 and was marginally reduced (*P*<0.06) in T18 CVS compared to that of control averages (Fig. 6c). Serum hCG heterodimer levels negatively correlated (r = -0.37) with LHCGR M_r _80 K protein expression in DS pregnancies.

## Discussion

In this study we have demonstrated that despite the high concentrations of serum hCG heterodimer and hCG β in DS pregnancies, their autocrine/paracrine effects on the placenta may be severely impaired due to a reduced expression of the hormone's cognate functional receptor. The accumulation of high levels of serum hormone or cytokine as a result of inadequate receptor-mediated signaling is not unprecedented [34,35]. Serum concentrations of IFN-γ are significantly higher in children suffering from innocuous mycobacterial infection due to the inheritance of non-functional IFN-γ R1 and 2 receptors [34,35]. Partial or complete inactivation of the *LHCGR *gene due to a naturally occurring somatic mutation within the coding sequence could be responsible for the increase in serum LH concentrations in leydig cell hypoplasia, male hypogonadism, and primary amenorrhea [49,50].

There are conflicting reports on the transcriptional regulation of human hCG α and β mRNA in DS pregnancies. For example, some studies suggest that the steady-state RNA synthesis from the *CGB *gene in Down's syndrome and gestation age-matched control pregnancies are comparable [51], while others show that the *CGB *gene is activated [52,53] or repressed [54] in comparisons of *in vitro *cultured trophoblasts from DS and control placenta.

The work presented here shows that when mRNA synthesis is quantified for a large number of CVS samples (41), the *CGB *(hCG β) gene in DS placenta is upregulated. This suggests that the conflict between previous reports may be due to sample size. In addition to this, the gestational age of the DS placenta, as well as the methodology employed by different laboratories to measure mRNA (northern blotting and PCR), and to purify and *in vitro *culture of cytotrophoblasts (CT) (discussed by Goshen [55]), could have contributed to the conflicting results. Purified CTs or placental explants have limited ability to differentiate under normal O_2 _tension and exhibit an invasive phenotype *in vitro *[54,56-58]. Purified CTs cultured under 2% O_2 _tension undergo a change into an invasive phenotype [59]. Notably, differentiation also leads to the formation of multinucleated giant cells instead of polarized epithelial layers of ST (the major source of hCG β) with typical microvillous structure and special antigen repertoire (Susan Fisher, personal communication).

The steady state level of transcription (as measured by Q- PCR of exons 1–5) from the *LHCGR *gene in DS was significantly higher than that of control CVS, whereas, the receptor expression in T18 and T13 pregnancies did not significantly differ from that of gestation age-matched control pregnancies (Fig. 2b). Nevertheless, a comparison of the expression of exon 11 from control and DS CVS indicated that a large proportion of the *LHCGR *transcripts in DS CVS may not be full-length, since they did not contain parts of exon 11. This might explain why up-regulation of *LHCGR *mRNA (exon 1–5) did not correlate with the expression of mature LHCGR p80 and other high M_r _isoforms which are less abundant in DS placenta (Figs. 2 and 6). These results also provide an explanation for previously reported increase in *LHCGR *mRNAs from T21 and T18 pregnancies compared to controls where cDNA common to all spliced variants of *LHCGR *mRNAs was used as an *in situ *hybridization probe on placental sections [60]. Interestingly, semi-quantitative PCR amplification of placental cDNA (*LHCGR *exons 9–11) and agarose gel analysis revealed that truncated products were highly abundant in late, compared to early, pregnancies (unpublished data).

Our protein data differ from those of others [60] who have demonstrated a significant increase in LHCGR protein in T21 and T18 placentas. However, this apparent contradiction is reconciled by considering that immunohistochemical staining of tissue sections using a polyclonal primary antibody raised against the common amino-terminal 15–38 residues of the LHCGR peptide [60] would stain both mature LHCGR and non-transducing LHCGR isoforms produced from alternatively spliced *LHCGR *mRNAs that have common N-terminal sequences (Figure 2, and [20,21,42,43]). Therefore, immunohistochemistry might be insufficient to distinguish mature LHCGR from its truncated isoforms (Fig. 6).

Alternative promoter use [20,61], and differential splicing of mRNA to produce various mRNA species [21,41-43,62] and multiple protein isoforms (Figs. 5 and 6, and [27,28,48]) are the hallmarks of molecular regulation of the *LHCGR *gene. While our work implies that there could be an increase in transcriptional initiation from the *LHCGR *gene in DS placenta, further work is needed to establish whether different promoters are utilized in DS compared to physiologically normal pregnancies. This could be important because in the transgenic mouse model developed by Huhtaniemi's group [61] there appears to be a link between alternative promoter utilization and differential splicing in transgenes. It is interesting to note that the multiple LHCGR protein isoforms (Figs. 5 and 6, and [28]) detected in placental extracts by Western blot are also expressed in LH-induced human neuroblastoma cells [48]. How many of these isoforms are capable of sequestering hCG by ligand binding is currently under investigation.

The clinical relevance of this report stems from the significance of hCG in establishing and maintaining placental/fetal development in human pregnancy. Given the recent discovery of an unanticipated role for LH/hCG in the distribution of cerebral blood flow [63], neurosteroidogenesis and fetal development of sensory and autonomic functions [64], the reduced expression of functional LHCGR protein in placenta may have far-reaching consequences. Indeed, the pathological activation of cerebral microglial cells abundantly expressing LHCGR, has been linked to Alzheimer's and other neurodegenerative diseases with high circulating LH [48,65]. Some outstanding questions remain to be answered, including whether the reduced expression of the functional LHCGR isoforms in placenta described here reflects a similar reduced expression in the fetal brain that might affect sensory and autonomic development in DS babies, and whether the reduction in functional LHCGR expression can be attributed to somatic mutations or an extra copy of chromosome 21.
